# Socrates’ “maieutics” and the ethical foundations of psychotherapy

**DOI:** 10.1192/j.eurpsy.2022.1927

**Published:** 2022-09-01

**Authors:** O. Doerr

**Affiliations:** Diego Portales University, Center Of Studies On Phenomenology And Psychiatry, Santiago, Chile

**Keywords:** Psychotherapy, Plato’s Dialogue Charmides

## Abstract

**Introduction:**

The noun “maieutics” derives from maia (mother, midwife) and the related verbs “maieusis” and “maieonuai” mean “giving birth” and “easing childbirth”. Socrates’ maieutics aspires to give birth to the truth in the youth. Since homeric times psychotherapy has been part of medical act. Initially, the physician’s word had a magical character. Plato rationalized this in many of his dialogues, specially in “Charmides”.

**Objectives:**

The search of the essential characteristics of this therapeutic method described by Plato, as well as its ethical implications

**Methods:**

Hermeneutic method

**Results:**

The consequences for doctor-patient relationship in general and psychotherapy in particular are: 1. Remedy and “epodé” (charm) must be applied in every doctor-patient relationship. 2. The body can only be healed if the soul is cured first by a charm. 3. The openness of the patient’s soul to the physician and the physician’s beautiful speech to the patient will enable the latter to reach the state of “sophrosyne” (temperance), condition of possibility of true health.

**Conclusions:**

In the discussion of the meaning of “sophrosyne”, Socrates questions disciples’ propositions and concludes that the only thing one can be sure of is that “sophrosyne” is a way of searching virtue (arete). Later, in Theaetetus, Plato adds another element: temperance is a “homoiosis theó”, that is, the assimilations of patient to God. With this Plato seals the ethical character of psychotherapy for ever.

**Disclosure:**

No significant relationships.

